# Dynamics of archaeal community in soil with application of composted tannery sludge

**DOI:** 10.1038/s41598-019-43478-y

**Published:** 2019-05-14

**Authors:** Ana Roberta Lima Miranda, Lucas William Mendes, Leandro Nascimento Lemos, Jadson Emanuel Lopes Antunes, Marineide Rodrigues Amorim, Vania Maria Maciel Melo, Wanderley Jose de Melo, Paul J. Van den Brink, Ademir Sergio Ferreira Araujo

**Affiliations:** 1 Federal University of Piauí, Department of Agricultural Engineering and Soil Science, Teresina, 64049-550 Brazil; 2 Laboratory of Molecular Ecology, CENA-USP, Piracicaba, SP Brazil; 3 Federal University of Ceará, Lembiotech, Fortaleza, Brazil; 4 São Paulo State University (Unesp), School of Agricultural and Veterinarian Sciences, Path of Access Prof. Paulo Donato Castellane, Km 5, Postal Code: 14884-900, Jaboticabal, SP Brazil; 5 University of Brazil: University campus Descalvado - Hilário da Silva Passos Avenue, 950 - University park, Descalvado, SP Brazil; 6 Wageningen University, Aquatic Ecology and Water Quality Management Group, P.O. Box 47, 6700 AA Wageningen, The Netherlands; 7 Wageningen Environmental Research, P.O. Box 47, 6700 AA Wageningen, The Netherlands

**Keywords:** Soil microbiology, Environmental impact

## Abstract

Application of composted tannery sludge (CTS) could promote a shift in the structure of soil microbial communities. Although the effect of CTS on bacterial community has been studied, it is unclear how the composition and diversity of archaeal community respond to CTS amendment and which environmental factors drive the community over time. Here, we hypothesize that the Archaea structure and composition respond to CTS amendment over the time. CTS had been previously applied annually along 6 years and this assessment occurred for 180 days following the application in the 7^th^ year by using different rates (0, 2.5, 5, 10 and 20 ton ha^−1^). We used amplicon 16S rRNA sequencing to assess the changes in the structure of the archaeal community. Thaumarchaeota and Euryarchaeota were the most abundant phyla found in soils with application of CTS, with Thaumarchaeota dominating the sequences in all samples with relative abundances of >98%. We observed a decreasing trend on the archaeal diversity over the time with increasing CTS application rate, together with an increase in the community similarity. The redundancy analyses (RDA) explained 43% of the total variation in operational taxonomic units and identified Na, pH, Cr and P as the main drivers of the archaeal community over time after application of highest CTS rates. CTS application changes the structure of Archaea community, with significant increase of Thaumarchaeota and Aenigmarchaeota groups, which can be further explored for its biotechnological use in contaminated soils.

## Introduction

Tannery sludge (TS) is an organic waste, produced in high amount, by tannery industries and contains high concentrations of salts, carbonates, hydroxides and chromium (Cr)^1^. Usually, this waste is disposed in the soil without previous treatment and, due to its chemical properties, could contribute to environmental pollution by affecting the soil properties, mainly biological ones^2^. Therefore, it is necessary to find suitable and efficient methods for TS treatment before its disposal in soil. Previous studies have proposed composting as a potential method for treating wastes before their disposal in soils^3–6^. However, the application of composted wastes could change the soil chemical and microbiological properties as well^5^.

Indeed, the application of composted wastes has effects on soil chemical properties. The application of composted tannery sludge (CTS) increases the Cr concentration, salinity and alkalinity^1,6^. The shifts in chemical properties could influence the soil microbial communities and consequently affect the biological processes in soil.

The knowledge about the factors driving the composition of microbial community in soil after application of waste is important to predict the potential effects on soil functioning since soil microorganisms play important roles in soil ecosystem^7^. Archaea community plays some fundamental ecological functions in the soil, including oxidation or reduction of metals and alteration of chemical conditions in the environment^8^. Since the application of CTS promotes changes in the soil environment, such as increased soil pH, salinity, Cr and organic matter content^1^, it is important to assess the effect of this waste on archaeal community. Previous studies have already evaluated the effect of tannery sludge on fungal and bacterial communities and revealed that these microorganisms are influenced by both characteristics of the waste and soil chemical properties^9–11^, but there are no studies focusing on effect of CTS on archaeal community. Considering the importance of Archaea in nitrogen and carbon turnover, and also their relationship with bacterial and fungal growth rates^12^, we addressed the following scientific questions in this paper: (1) how do the composition and diversity of archaeal community respond to CTS amendment over time? (2) which environmental factors drive the composition the Archaea community over time?

## Results

**Figure 1:**
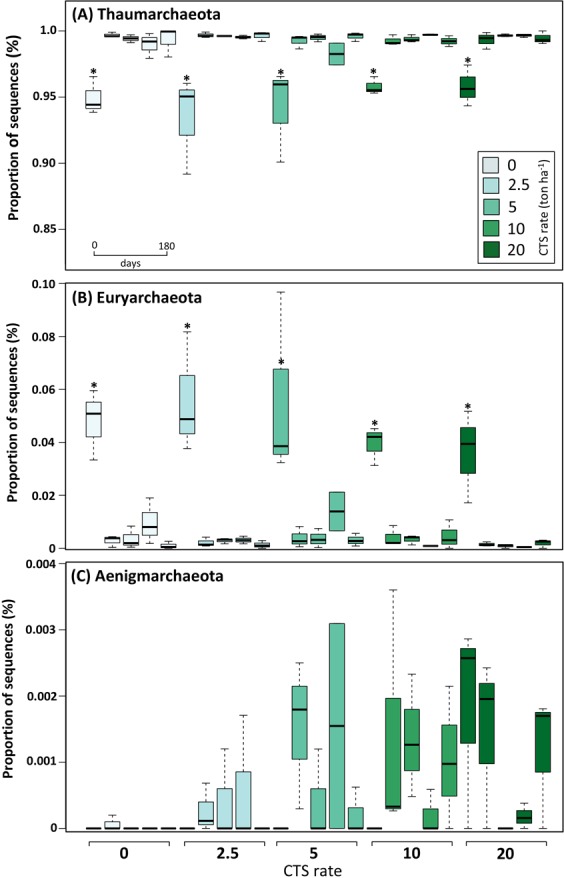
Distribution of the most abundant archaeal phyla between treatments and time after application of different rates of composted tannery sludge (CTS). Center lines in the boxplots show the medians; box limits indicate the 25^th^ and 75^th^ percentiles. Asterisks indicate significant differences within a treatment based on Tukey’s test (P < 0.05).

CTS promoted significant increases in pH, total organic C (TOC), Cr and the content of total organic C, P, Ca, and Mg (Table S1). Cr, TOC, P and Ca increased significantly at all CTS rates. Through Next-Generation Sequencing (NGS) we assessed the structure and diversity of the archaeal community and, after the sequencing pre-processing, more than 360,000 quality sequences were used and grouped in 194 Operational Taxonomic Units (OTU), affiliated to five phyla and 10 classes. The most abundant phyla were Thaumarchaeota (98.5% of the total sequences), followed by Euryarchaeota (1.3%), Woesearchaeota (0.2%) and Aenigmarchaeota (0.05%). The response of the main archaeal phyla over time showed an increase in the relative abundance of Thaumarchaeota and a decrease in the relative abundance of Euryarchaeota; also Aenigmarchaeota increased with the CTS amendment (Fig. 1).

**Figure 2:**
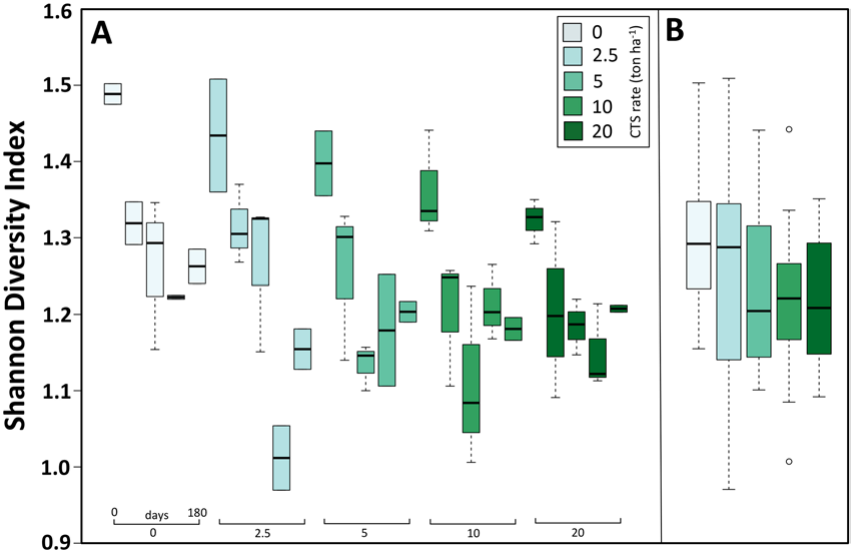
Shannon’s diversity index based on archaeal 16S rRNA genes of soil treated with composted tannery sludge (CTS) at different rates. (**A**) Variation of archaeal diversity according to time (0 to 180 days) after CTS application. (**B**) Average diversity for each treatment. Center lines in the boxplots show the medians; box limits indicate the 25^th^ and 75^th^ percentiles.

We observed a decreasing diversity over the time (Fig. 2A) and with the increased CTS application (Fig. 2B). An increase in the archaeal community similarity was observed with increasing CTS rate (Fig. S1), showing a homogenization of the community diversity.

**Figure 3:**
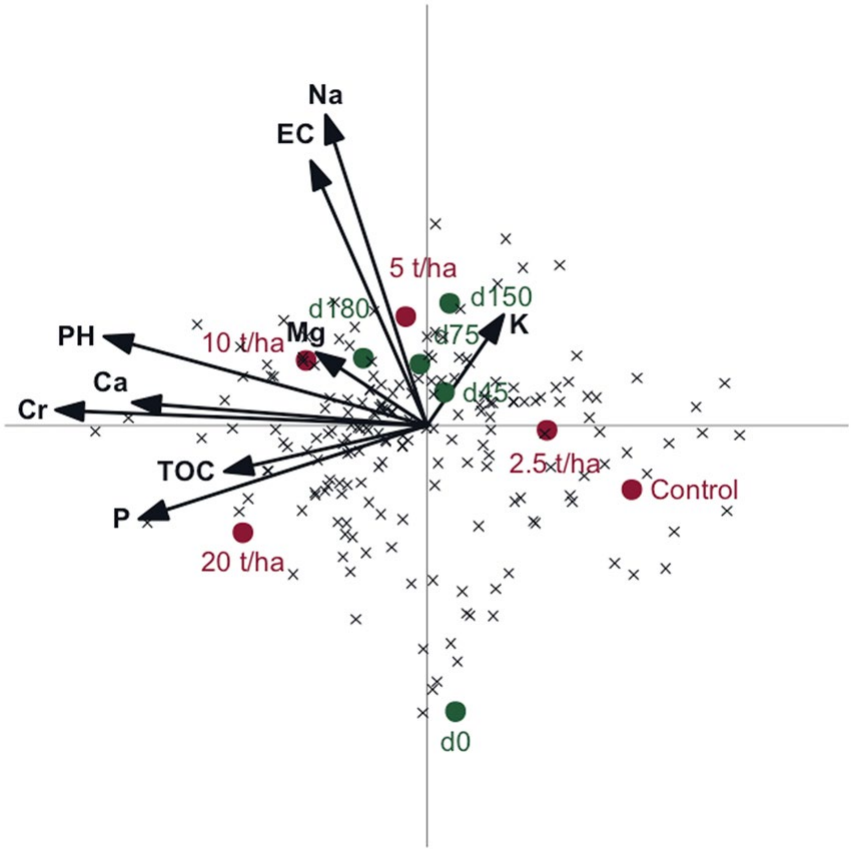
Redundancy analysis diagram (RDA) of correlations between significant physico-chemical proprieties and OTU’s. (**A**) Rates of composted tannery sludge (CTS - 2.5, 5, 10 and 20 ton/ha); Time of sampling in days (0, 45, 75, 150 and 180); Total organic carbon – TOC; P – phosphorus; Ca – calcium; Cr – Chromium; pH – soil pH; EC – electric conductivity. All OTU’s are denoted by a small x-mark.

Physico-chemical properties explained 43% of the total variation in community structure based on OTU level, where 65% and 15% were displayed on the first (horizontal) and second (vertical) axes, respectively (Fig. 3). Treatments and sampling period were used as supplementary environmental variables. Soil samples were separated according to the treatments, with a clear pattern from the unamended soil to amended soils with increased CTS rate application, as confirmed by ANOSIM analysis (Table S2). This result revealed Na, pH, Cr and P as the most important factors driving the archaeal community composition over time.

**Figure 4:**
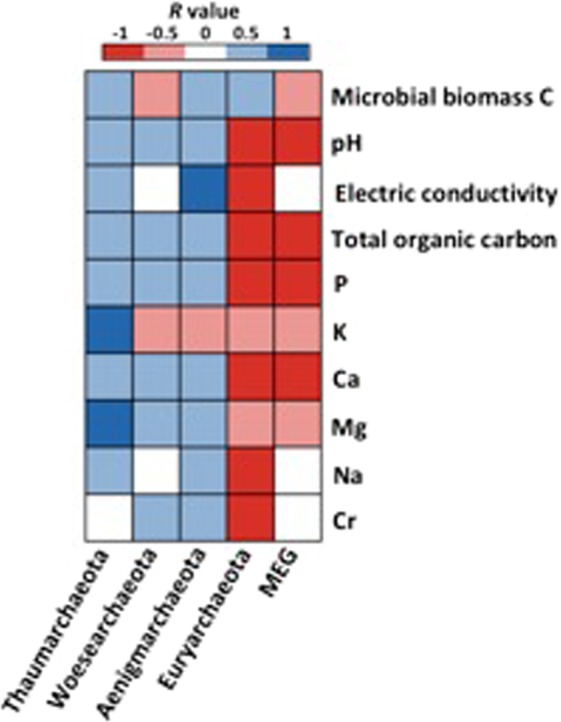
Heatmap showing the Spearman’s rank correlation coefficients between phyla abundance relative to soil factors. Blue and red colors indicate significant positive and negative correlations, respectively (P < 0.05); white indicate no significant correlation (P > 0.05).

To further explore the correlation between archaeal phyla and soil chemical parameters, we calculated all possible Spearman’s rank correlation (Fig. 4). Thaumarchaeota, Woesearchaeota and Aenigmarchaeota showed a positive correlation with most of the variables while Euryarchaeota and MEG (Miscellaneous Euryarchaeotic Group) correlated negatively.

The Unweighted UniFrac distance metric with Principal Coordinates Analysis (PcoA) clustered the samples according to the CTS rate (Fig. S2). A pairwise comparison at OTU level between each treatment with the control revealed that the increased rate of CTS application increased the number of OTUs, where T2 presented three OTUs with increased abundance, followed by T3 with 15 OTUs, T4 with 22 and T5 with 23 OTUs with increased abundance in comparison with the control (Fig. S3).

**Figure 5:**
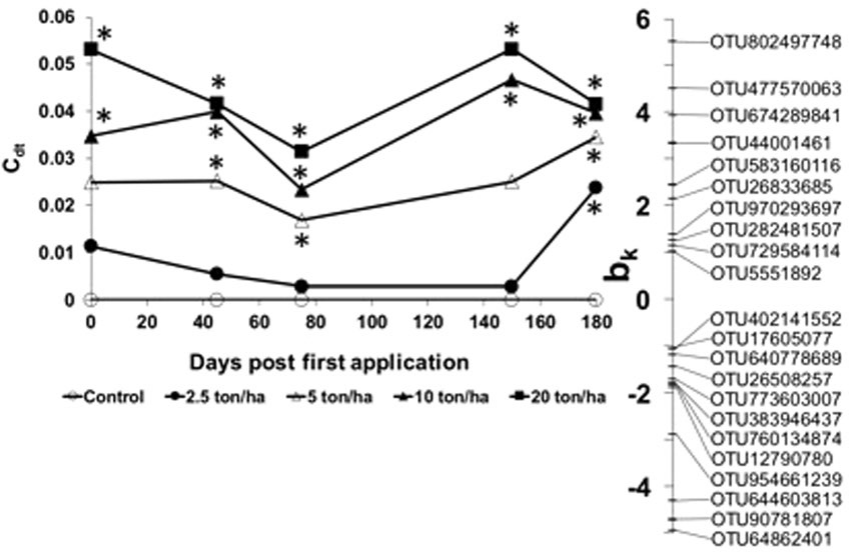
Principal response curve (PRC) diagram of OTU data set indicating the effects of the composted tannery sludge (CTS) into the soil. The lines represent the course of the treatment levels in time. The OTU weight (b_k_) can be interpreted as the affinity of the OTU’s with the PRC. The Monte Carlo permutation test indicated that a significant part of the variance explained by treatment is displayed in the diagram (p = 0.014). Only OTUs with a b_k_ value >1 or <−1 are shown for clarity. Asterisks indicate which treatments are significantly different from the controls (Williams test on PCA coordinates, see materials and methods section).

The principal response curve (PRC) analysis at OTU level showed 10% of the total variance being attributed to the sampling time, while 45% was attributed to CTS rates (Fig. 5). Two OTUs presented a high positive response to the treatment (OTU802497748 and OTU477570063) with *b*_*k*_ greater than 4; while three OTUs showed a high negative response (OTU64862401, OTU90781807, and OTU644603813) with *b*_*k*_ lower than −4. Blast was used to better identify each responsive OTU showing extreme *b*_*k*_ values in the PRC analysis and all of them belong to uncultured archaeon affiliated to the Thaumarchaeota phylum (Table S3).

Co-occurrence network analysis showed an increase in complexity from T1 to T4, with a relatively low complexity in T5 (Fig. S4). The T4 treatment presents the highest complexity, with 165 correlations, and average degree of 9.16 (Table S4).

## Discussion

We assessed the structure and diversity of archaeal community in response to application of CTS for seven years in soil. For this, we sequenced the V4 region of the 16S rRNA gene to describe patterns in taxonomical composition of soil archaeal communities and correlated them with CTS induced changes in soil parameters. As expected, Thaumarchaeota and Euryarchaeota were the most abundant phyla found in soils with application of CTS (Fig. 1), which corroborates previous studies that reported these phyla as dominant in several soils^13–15^. Interestingly, the phylum Thaumarchaeota was dominant in all samples and matches the fact that this phylum is found in high abundance in both marine and soils environments^16,17^. Members of this phylum play important roles in biogeochemical cycles, such as nitrogen and carbon^18^. This phylum represents a group of organisms that are able to oxidize ammonia aerobically and is important for the nitrification process in soils^19^. Considering that nitrogen is a key element controlling terrestrial productivity, the assessment of the effects of CTS on this community is of paramount importance.

In line with our first hypothesis, the relative abundance of archaeal phyla showed different responses to CTS amendment. The phylum Thaumarchaeota increased in relative abundance over time, while Euryarchaeota decreased (Fig. 1) as a response to short-term CTS application (180 days) in the seventh consecutive year. Probably, this increasing abundance is associated with the increase in soil pH over time (Table S2). Soil pH is the main driver of the abundance of Thaumarchaeota and this phylum contains groups adapted to different pH range, with *Nitrososphaera* as the group found under alkaline conditions^20^. In contrast, Euryarchaeota decreased probably as response to increasing Cr accumulation^21^, as confirmed by the strong negative correlation with CTS rate (Fig. 4). On the other hand, Aenigmarchaeota increased with increasing CTS rates, indicating that the waste stimulated the abundance of this phylum. This phylum was recently described^22^ and their members were found in wastewater from mines and sediments of hot springs^23^. Interestingly, they are adapted to extreme environments such as high pH variation, temperature and heavy metals^23^, which may explain the increase of this phylum in the samples with high CTS rates. Our results reinforce the observation that changes in soil chemical properties imposed by the CTS application have affected the different archaeal groups distinctly. The structure of archaeal communities is influenced by changing environmental factors, such as soil pH, moisture content, temperature and organic carbon, among others^24^.

The diversity of archaeal community suggests a detrimental effect of CTS, with a trend to decrease the diversity with the increase of CTS rate. The application of CTS changed some soil chemical properties, herewith selecting specific groups of Archaea, resulting in more homogenous communities with increased CTS rate (Fig. S1B)^25^.

The redundancy analysis (RDA) indicates that changes in soil chemical parameters over time due to CTS application resulted in changes in the composition of the archaeal community (Fig. 5). This finding is in agreement with previous studies that have shown that the archaeal communities are significantly affected by environmental variables, such as Na^26^, P^27^, pH^28^ and Cr^21^. Thus, our results confirm that different environmental drivers affect different groups of Archaea. Considering that most of the sequences were affiliated to Thaumarchaeota phylum, we suggest that the effects of CTS application may be better characterized at the phylogenetic level. Recently, Gubryn-Rangin *et al*.^29^ demonstrated links between the phylogeny of soil Thaumarchaeota and pH. The analysis based on the Unifrac distance corroborates our previous analysis revealing a differentiation of the archaeal community at the phylogenetic level, *i*.*e*. a difference of the species within the phylum Thaumarchaeota. In a study with 46 soils presenting 29 soils parameters, Oton *et al*.^30^ showed that adaptation to pH and organic matter content induced strong ecological coherence at various taxonomic level resolutions for Thaumarchaeota. Indeed, a pairwise comparison in our data revealed that the increased rate of CTS increased the number of OTUs that were selected and increased in relative abundance (Fig. S3).

As an important method for evaluating the response of community composition over time, the PRC method is a multivariate technique based on the redundancy analysis ordination technique. The PRC analysis pointed to specific OTUs that responded positively and negatively to the treatments compared with the control as they develop over time. Thus, it shows a pattern of archaeal OTUs presenting greater or lower sensitivity to CTS. This result resembles the effect of CTS on bacterial community as previously reported by Miranda *et al*.^9^. It means that the CTS amendment increased the abundance of specific OTUs that could show adaptation to pollution, i.e. high concentration of Cr, as reported before.

Finally, the co-occurrence network analysis showed that the network complexity increased with increasing CTS application rate (Fig. S4). The network analysis revealed that the CTS application directly affected the dynamics of the archaeal community, mainly the Thaumarchaeota group, by increasing the number of interaction among them. These effects on the community dynamics may be explained by the increased abundance of specific organisms (defined here at OTU level). We also identified the archaeal OTUs with more betweeness centrality, which may represent key taxa for a connected community^31^. Interestingly, for all the networks, the key OTU was affiliated to the genus *Nitrososphaera* (Table S5). This genus belongs to the group of ammonia oxidizing Archaea, an important player in the transformation of nitrogen^32^. Also, *Nitrososphaera* presents high metabolic versatility and can adapt to different environmental conditions^33^. A previous study has found biochemical characteristics of *Nitrososphaera*, such as chromate reductase activity, that contribute to its resistance to Cr^34^. This could explain the key role of this genus in the community dynamics in CTS-treated soils with high accumulation of Cr (Fig. S4 and Table S5). A genomic analysis of the species *Nitrosphaera gargensis* revealed the presence of two NADPH-dependent FMN reductases and four nitroreductases that could have a chromate reductase activity, which are suggested to be determinants of chromate resistance in bacteria^33,34^. In this sense, further studies could explore the role of specific members of Thaumarchaeota in bioremediation of soils contaminated with high rates of chromium.

Our results indicated a CTS-induced alteration of the archaeal communities with a decreasing diversity and increasing community similarity with CTS application rates. More specifically, we demonstrated that the phyla Thaumarchaeota and Aenigmarchaeota were the most responsive groups, which are able to resist to and potentially oxide contaminants, and can be further used as biotechnological products for contaminated sites.

## Methods

This study was performed at the Federal University of Piauí, Brazil. CTS was obtained by mixing TS with sugarcane straw and cattle manure (ratio 1:3:1; v:v:v) after a process of composting over three months. The properties of CTS were assessed according APHA^35^ and USEPA^36^ (Table S6). This field experiment presents a history of CTS application, one time per year, for 6 years (2009–2014) in five doses: 0 (without CTS application), 2.5, 5, 10, and 20 ton/ha of CTS (dry basis). Detailed information of this experiment with CTS can be found in Sousa *et al*.^37^.

In 2015 (7^th^ year of application), CTS was applied on the soil surface and incorporated into the 0–20 cm layer with a harrow. In order to measure the time-dependent effect of CTS on the diversity and structure of archaeal community, soils were sampled at 0, 45, 75, 150 and 180 days after CTS application from January to June, 2015. Thus, four replicates were sampled in each plot (0–20 cm), sieved (2 mm), and stored at −20 °C. The chemical properties of the soil (Table S1) were determined according to methods described in Embrapa^38^.

To assess the archaeal community profile we used the same procedures as described in Araujo *et al*.^15^, as follow: soil DNA was extracted from 0.5 g (field moist soil) of soil using the PowerLyzer PowerSoil DNA Isolation Kit (MoBIO Laboratories, Carlsbad, CA, USA), according to the manufacturer’s instructions. The DNA extraction was performed in triplicate for each soil sample. The quality and concentration of the extracted DNA was estimated using a Nanodrop 1000 (Thermo Scientific, Waltham, MA, USA). The amplicon library of the archaeal 16S rRNA genes was amplified using the V4 region-specific primers 515F (5′-GTGCCAGCMGCCGCGGTAA-3′) and 806R (5′-GGACTACHVGGGTWTCTAAT-3′)^39^. The first step amplification comprised 25 μL reaction containing the following solutions: 14.8 μL of nuclease-free water (Certified Nuclease-free, Promega, Madison, WI, USA), 2.5 μL of 10X High Fidelity PCR Buffer (Invitrogen, Carlsbad, CA, USA), 1.0 μL of 50 mM MgSO_4_, 0.5 μL of each primer (10 μM concentration, 200 pM final concentration), 1.0 unit of Platinum Taq polymerase High Fidelity (Invitrogen, Carlsbad, CA, USA), and 4.0 μL of template DNA (10 ng). The conditions for PCR were as follows: 94 °C for 4 min to denature the DNA, with 25 cycles at 94 °C for 45 s, 60 °C for 60 s, and 72 °C for 2 min, with a final extension of 10 min at 72 °C. In the second step, a unique pair of Illumina Nextera XT indexes (Illumina, San Diego, CA) was added to both ends of the amplified products. Each 50 μL reaction contained the following solutions: 23.5 μL of nuclease-free water (Certified Nuclease-free, Promega, Madison, WI, USA), 5.0 μL of 10X High Fidelity PCR Buffer (Invitrogen, Carlsbad, CA, USA), 4.8 μL of 25 mM MgSO_4_, 1.5 μL of dNTP (10 mM each), 5.0 μL of each Nextera XT index (Illumina, San Diego, CA, USA), 1.0 unit of Platinum Taq polymerase High Fidelity (Invitrogen, Carlsbad, CA, USA), and 5.0 μL of each product from previous PCR. The conditions for this second round PCR were as follows: 95 °C for 3 min to denature the DNA, with 8 cycles at 95 °C for 30 s, 55 °C for 30 s, and 72 °C for 30 min, with a final extension of 5 min at 72 °C.

After indexing, the PCR products were cleaned up using Agencourt AMPure XP–PCR purification beads (Beckman Coulter, Brea, CA, USA), according to the manufacturer’s manual, and quantified using the dsDNA BR assay Kit (Invitrogen, Carlsbad, CA, USA) on a Qubit 2.0 fluorometer (Invitrogen, Carlsbad, CA, USA). Once quantified, different volumes of each library were pooled into a single tube in equimolar concentration. After quantification, the molarity of the pool was determined and diluted to 2 nM, denatured, and then diluted to a final concentration of 8.0 pM with a 20% PhiX (Illumina, San Diego, CA, USA) spike for loading into the Illumina MiSeq sequencing device (Illumina, San Diego, CA, USA).

Sequence data were processed using QIIME 1.0 following the UPARSE standard pipeline according to Brazilian Microbiome Project^40^ to produce an OTU table and a set of representative sequences. Briefly, the reads were truncated at 130 bp and quality-filtered using a maximum expected error value of 1. Pre-filtered reads were dereplicated and singletons were removed using USEARCH 7.0. These sequences were clustered into OTUs at a 97% similarity cutoff following the UPARSE pipeline^41^. After clustering, the sequences were aligned and taxonomically assigned against the Silva database^42^.

We used analysis of similarity ANOSIM to test whether the treatments harbored significantly different microbial community structure. Alpha diversity was calculated from a matrix of richness, using the Shannon index. ANOSIM and alpha diversity index were calculated with the software PAST 3^43^. Further, in order to test the significance of the treatment in clustering of the samples, we analyzed the community structure based on the OTU phylogenetic distance using unweight UniFrac dissimilarity^44^.

In order to visualize the differential archaeal community composition among treatments, the Statistical Analysis of Metagenomic Profiles (STAMP) software was used^45^. For this, the OTU table generated by QIIME was used as input and the P-values were calculated using the ANOVA with post-hoc test Tukey-Kramer, followed by Benjamini-Hochberg false discovery rate correction^46^. The analysis of similarity (SIMPER) was used to evaluate the community similarity within/ between treatments and time using the software Primer6 (PrimerE, Ivybridge, United Kingdom). Spearman’s rank correlation coefficient were calculated to explore the relationship between archaeal community with environmental factors according to the different treatment using the ‘multtest’ package in R^47^ and the correction was made using Benjamini-Hochberg false discovery rate. For visualization, heatmaps were constructed based on z-score transformed phylum abundance to improve normality and homogeneity of the variances. The z-score was calculated as the deviation from row mean in units of standard deviations above or below the mean.

In order to assess the complexity of the interactions among archaeal taxa, we performed network analysis according to Mendes *et al*.^48^. Briefly, non-random co-occurrence analyses were performed using SparCC^49^ based on a OTU table clustered at 97% identity. For each network, P-values were obtained by 99 permutations of random selections of the data table, subjected to the same analytical pipeline. In the networks, we included SparCC correlations statistically significant (P < 0.01) and with a magnitude of >0.7 or <−0.7. The nodes in the reconstructed network represent taxa at OTU level, whereas the edges represent significantly positive or negative correlations between nodes. The network graphs were made based on a set of measures, as number of nodes, number of edges, modularity, number of communities, average node connectivity, average path length, diameter and cumulative degree distribution. Co-occurrence analyses were carried out using the Python module ‘SparCC’ and networks visualization and properties measurements were calculated with the interactive platform Gephi^50^.

The structure of the Archaea community (as OTU’s) in the soil samples and its correlation with biological and environmental parameters was determined using Redundancy Analysis (RDA; length of gradient = 1.6). Since we analyzed relative abundance data, the data were arcsine transformed before analysis. The significance of each physico-chemical parameters for explaining the variation in community composition was tested with Monte Carlo permutation tests using sampling date as blocks. For data set an RDA analysis was performed using the significant physico-chemical parameters as explanatory variables and treatment and sampling date as passive explanatory variables. In addition, the dataset at the OTU level was analyzed by the principal response curve (PRC) method^51^ to show and test temporal changes in archaeal community of the soil caused by different rates of CTS as compared with those in the control (CTS-free soil) and also to identify specific taxa that responded positively or negatively to CTS application. The RDA and PRC analyses were performed using the CANOCO Software package, version 5^52^.

The overall significance of the CTS treatment regime on the variation in OTU (p ≤ 0.05) was tested by performing 999 Monte Carlo permutations^53^. The NOECs of the tannery sludge treatment per sampling date was calculated by applying the Williams test^54^ to the sample scores of the first principal component of each sampling date^53^, using the Community Analysis computer program, version 4.3.05^55^ using a significance level of 0.05^56^.

## Supplementary information

Suplementary Material
