# Supplementary material for: Dynamics of archaeal community in soil with application of composted tannery sludge
Source: Sci Rep. 2019 May 14;9:7347. doi: 10.1038/s41598-019-43478-y (PMC6517401; doi:10.1038/s41598-019-43478-y)
Supplement: Supplementary file 1 — Suplementary Material [file 41598_2019_43478_MOESM1_ESM.pdf]

SUPPLEMENTARY MATERIAL

Dynamics of archaeal community in soil with application of composted tannery sludge

Ana Roberta Lima Miranda, Lucas William Mendes, Leandro Nascimento Lemos, Jadson Emanuel Lopes Antunes, Marineide Rodrigues Amorim, Vania Maria Maciel Melo, Wanderley Jose de Melo, Paul J. Van den Brink, and Ademir Sergio Ferreira Araujo

Supplementary Tables 1 to 6

Supplementary Figures 1 to 4

SUPPLEMENTARY TABLES

Supplementary Table S1. Chemical properties of the soil after eight years of consecutive application of composted tannery sludge (CTS).

CTS	pH	EC[1]	TOC[2]	P	K	Ca	Mg	Na	Cr
(ton ha^{-1})	$CaCl_2$	dS m^{-1}	g kg^{-1}	mg dm^{-3}	--------mmolc dm^{-3}--------				Mg kg^{-1}
0	5.1 b	0.5 a	4.9 c	4.3 d	1.9 a	12.5 c	5.0 b	4.4 a	5.8 e
2.5	5.4 b	0.5 a	5.7 b	5.0 c	1.8 a	17.0 b	5.8 b	4.9 a	27.2 d
5	5.8 a	0.5 a	6.8 a	6.0 c	1.9 a	22.8 a	7.0 a	4.9 a	58.0 c
10	6.2 a	0.6 a	6.6 a	7.8 b	1.9 a	23.5 a	7.5 a	4.6 a	96.6 b
20	6.6 a	0.6 a	7.1 a	9.5 a	1.8 a	25.8 a	7.0 a	4.9 a	165.9 a

[1] Electric conductivity; [2] Total organic carbon. Means followed by the same letter in in each column are not significantly different (P<0.05) by Tukey test.

Supplementary Table S2. Results of ANOSIM based on Bray-Curtis dissimilarity index evaluating the variation in the composition and structure of archaeal community (at OTU level) among treatments with different doses of composted tannery sludge (CTS rate in Mg ha^{-1}). The abbreviations refer to the treatments: T1 = 0; T2 = 2.5; T3 = 5; T4 = 10; T5 = 20.

	***P*-value**	***R***
Treatments	0.0001	0.206
Pairwise comparison		
T1 vs. T2	0.8426	
T1 vs. T3	0.0019	
T1 vs. T4	0.0001	
T1 vs. T5	0.0001	
T2 vs. T3	0.0178	
T2 vs. T4	0.0003	
T2 vs. T5	0.0001	
T3 vs. T4	0.1658	
T3 vs. T5	0.0002	
T4 vs. T5	0.0005	

Supplementary Table S3. Best top hit of each OTU (Operational Taxonomic Units) identified in the PRC (Principal Response Curves) analysis.

OTU	Best-hit/Genbank accession	Identity(%) E-value	Environment	Reference
OTU802497748	Uncultured archaeon (JQ726432)	99/8e-58	Soil	Chakraborty et al. (2010)
OTU26833685	Uncultured archaeon (MH387273)	100/6e-59	Hot spring	Unpublished
OTU477570063	Uncultured archaeon (KT461144)	100/6e-59	Soil	Lynn et al. (2017)
OTU44001461	Uncultured archaeon (KY891231)	99/3e-57	Wetland	Unpublished
OTU64862401	Uncultured archaeon (MG430471)	99/3e-57	Soil	Unpublished
OTU583160116	Uncultured archaeon (KX687454)	99/3e-57	Sediment	Unpublished
OTU644603813	Uncultured archaeon (AB848867)	99/8e-58	Soil	Kim et al. (2017)
OTU773603007	Uncultured archaeon (KP328018)	100/6e-59	Soil	Yalong et al. (2016)
OTU954661239	Uncultured archaeon (KP018521)	99/3e-57	Soil	Zhang et al. (2017)
OTU12790780	Uncultured archaeon (KY802138)	100/6e-59	Wetland	Unpublished
OTU674289841	Uncultured archaeon(MH387271)	98/1e-55	Hot spring	Unpublished
OTU383946437	Uncultured archaeon (JN205364)	99/3e-57	Soil	Unpublished
OTU760134874	Uncultured archaeon (KY802232)	98/1e-55	Wetland	Unpublished
OTU90781807	Uncultured archaeon (KY802279)	100/6e-59	Wetland	Unpublished
OTU282481507	Uncultured archaeon (KP096905)	98/1e-55	Hyperalkaline springs	Quéméneur et al. (2015)
OTU26508257	Uncultured archaeon (FJ184925)	99/8e-58	Deep subsurface	Unpublished
OTU17605077	Uncultured archaeon KF276046)	99/3e-57	Soil	Hong et al. (2014)
OTU640778689	Uncultured archaeon (GQ126931)	99/3e-57	Soil	Unpublished
OTU729584114	Uncultured archaeon (KT216123)	98/1e-55	Soil	Unpublished
OTU5551892	Uncultured archaeon (MH313352)	98/6e-54	Sediment	Unpublished
OTU402141552	Uncultured archaeon (KY893105)	97/1e-56	Wetland	Unpublished
OTU970293697	Uncultured archaeon (KX687454)	98/6e-54	Sediment	Unpublished

Supplementary Table S4. Correlations and topological properties of the microbiome networks from archaeal communities in soil amended with CTS.

Network properties	T1	T2	T3	T4	T5
Number of nodes[a]	31	40	34	36	31
Number of edges[b]	76	105	82	165	75
Positive edges[c]	75	97	76	165	75
Negative edges[d]	1	8	6	0	0
Modularity[e]	0.523	0.561	0.547	0.217	0.433
Number of communities[f]	5	6	7	4	5
Network diameter[g]	5	7	8	4	5
Average path length[h]	2.35	3.07	3.27	1.82	2.52
Average degree[i]	4.90	5.25	4.82	9.16	4.83
Av. clust. coefficient[j]	0.569	0.592	0.612	0.670	0.568

[a]Microbial taxon (at genus level) with at least one significant ($P < 0.01$) and strong (SparCC > 0.7 or < -0.7) correlation;
[b]Number of connections/correlations obtained by SparCC analysis;
[c]SparCC positive correlation (> 0.7 with $P < 0.01$);
[d]SparCC negative correlation (< -0.7 with $P < 0.01$);
[e]The capability of the nodes to form highly connected communities, that is, a structure with high density of between nodes connections (inferred by Gephi);
[f]A community is defined as a group of nodes densely connected internally (Gephi);
[g]The longest distance between nodes in the network, measured in number of edges (Gephi);
[h]Average network distance between all pair of nodes or the average length off all edges in the network (Gephi);
[i]The average number of connections per node in the network, that is, the node connectivity (Gephi);
[j]How nodes are embedded in their neighborhood and the degree to which they tend to cluster together (Gephi).

Supplementary Table S5. OTUs with more betweeness centrality and number of correlations for each treatment.

Treatment	OTU Number	Phylum	Genus	Betweeness Centrality[a]	Degree[b]
**T1**	411653388	Thaumarchaeota	Nitrososphaera	59.6	10
**T2**	809177462	Thaumarchaeota	Nitrososphaera	197.5	10
**T3**	282481507	Thaumarchaeota	Nitrososphaera	156.4	6
**T4**	5551892	Thaumarchaeota	Nitrososphaera	56.7	22
**T5**	411653388	Thaumarchaeota	Nitrososphaera	164.9	13

[a]The fraction of cases in which a node lies on the shortest path between all pair of other nodes, interpreted as keystone species.

[b]Number of connections/correlations obtained by SparCC analysis;

Suplementary Table S6. Chemical attributes of the CTS used in the experiment.

pH	Moisture	TOC	N	P	K	Ca	Mg	Na	S	Cu	Ni	Cd	Cr	Pb
H_2O	%	---------------------g kg^{-1}--------------------								----------mg kg^{-1}----------				
7.5	68	201	15	4.9	2.9	121	7.2	49.1	10	16	23	1.9	1,943	40
MLP*	-	-	-	-	-	-	-	-	-	200	70	3	150	180

* Maximum limit permitted by Brazilian regulation (CONAMA, 2009).

\

SUPPLEMENTARY FIGURES

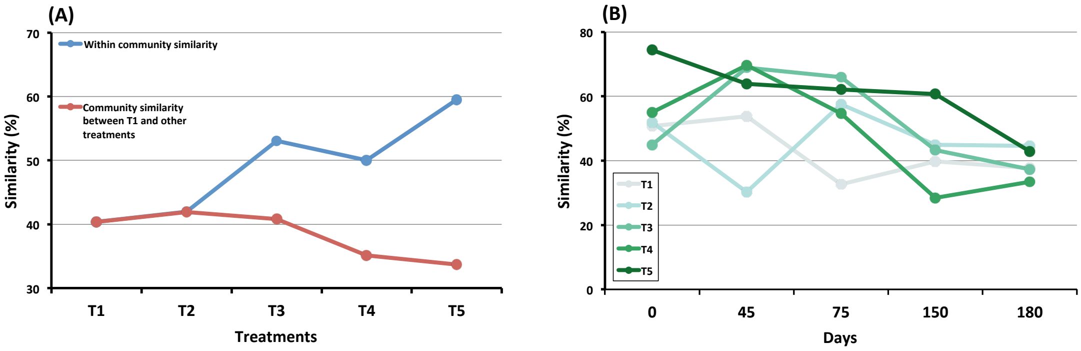

Supplementary Figure 1. Similarity percentage analysis (SIMPER) based on Bray-Curtis index of the archaeal community in soils with different rate of CTS amendment. **(A)** Similarity between treatments. **(B)** Similarity along the days of CTS application within each treatment.

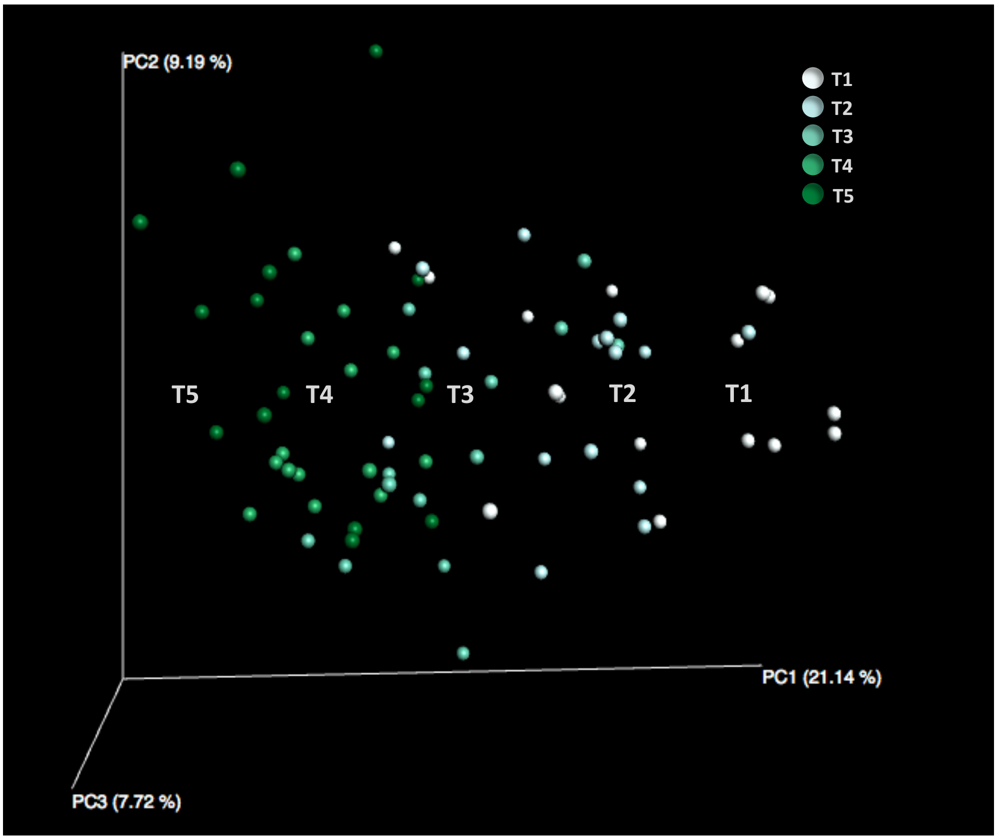

Supplementary Figure 2. Principal coordinate analysis based on the unweight unifrac distance of the archaeal communities from soils amended with different rates of CTS. T1 =

0 ton ha^{-1}; T2 = 2.5 ton ha^{-1}; T3 = 5 ton ha^{-1}; T4 = 10 ton ha^{-1}; T5 = 20 ton ha^{-1}.

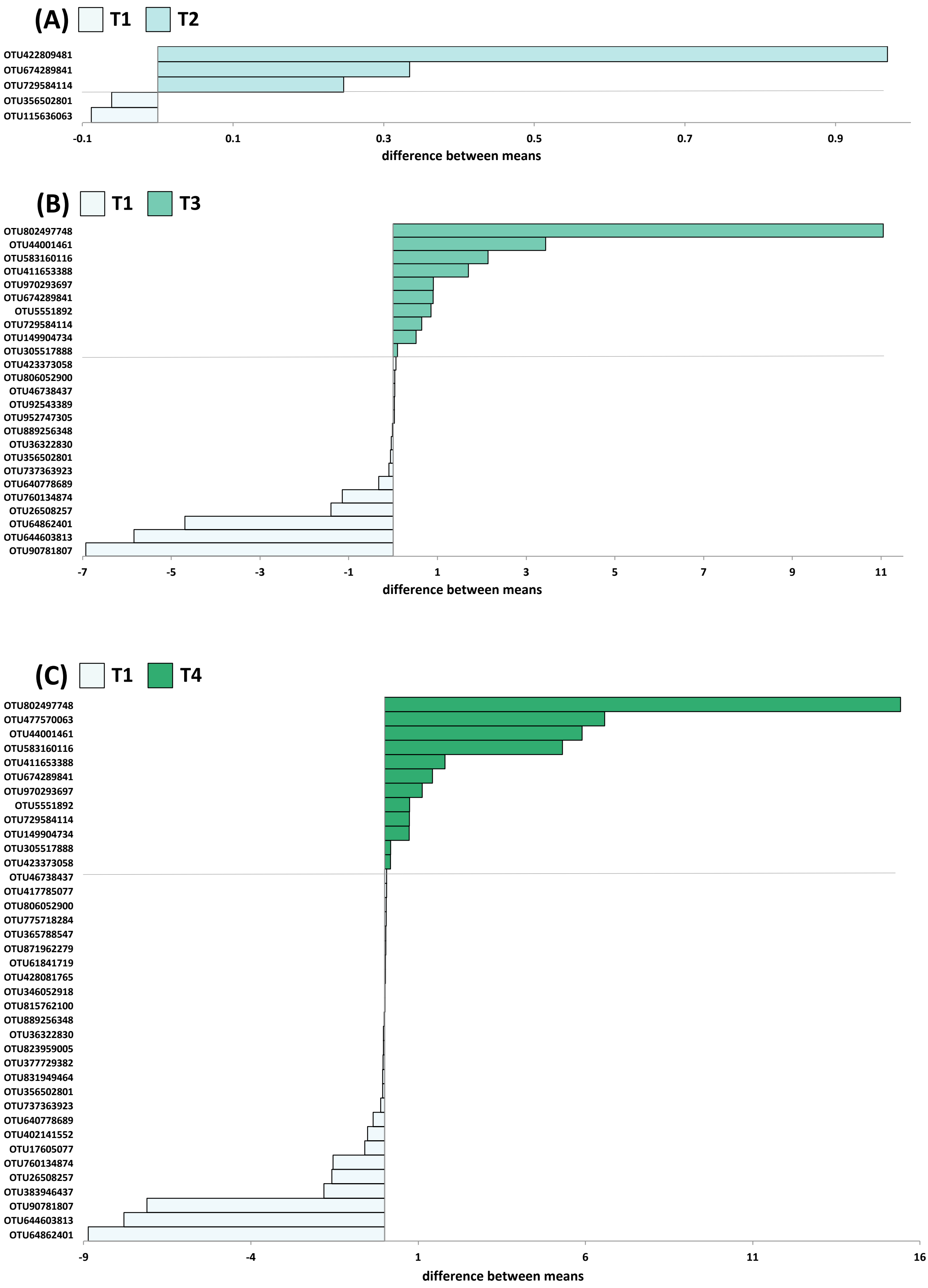

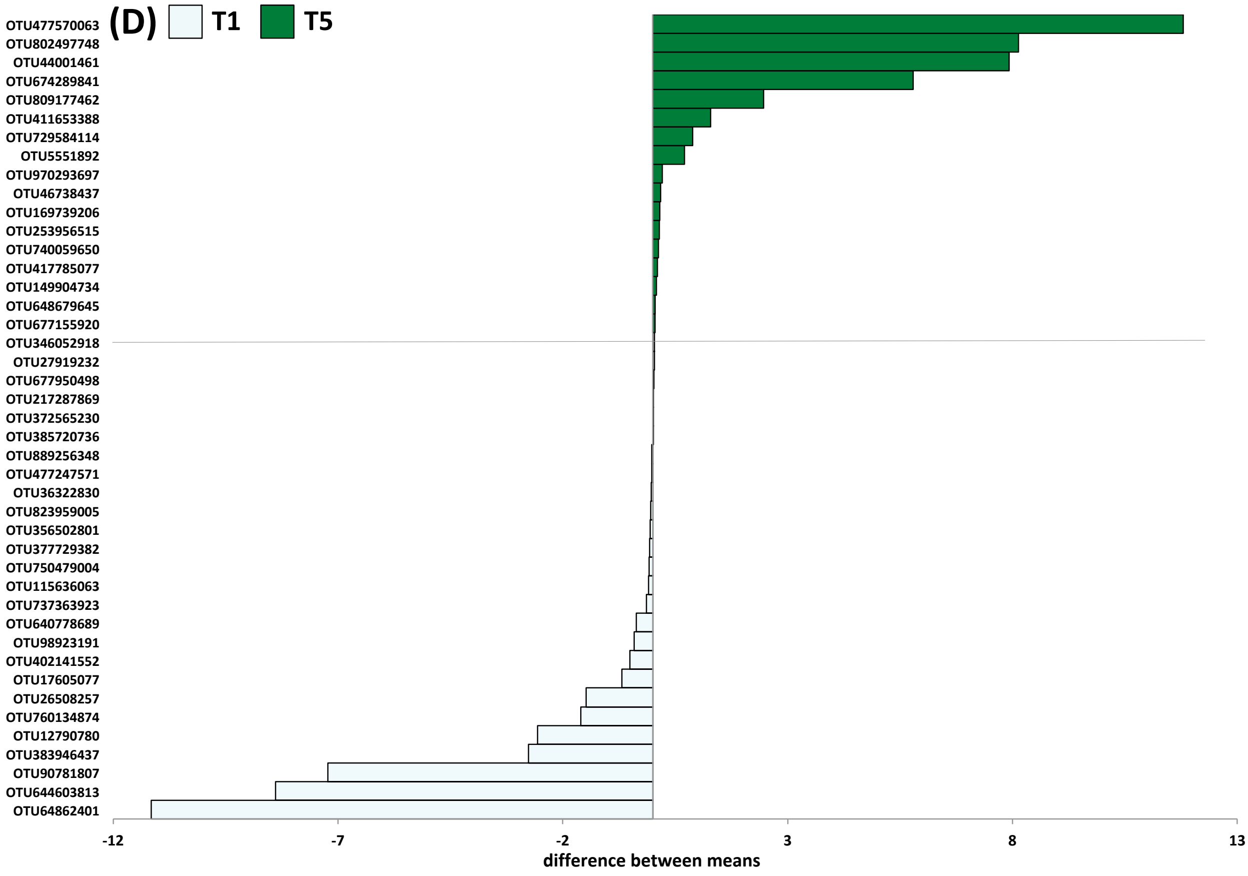

Supplementary Figure 3. Differential abundance of specific OTUs in response to CTS application in soils. Each bar represents the difference between mean of the OTU abundance comparing the different CTS treatment with the control. Only OTUs with significant difference are shown ($P < 0.05$). P values were calculated based on Welch's *t* test and corrected by Benjamini-Hochberg false discovery rate. . T1 = 0 ton ha^{-1}; T2 = 2.5 ton ha^{-1}; T3 = 5 ton ha^{-1}; T4 = 10 ton ha^{-1}; T5 = 20 ton ha^{-1}.

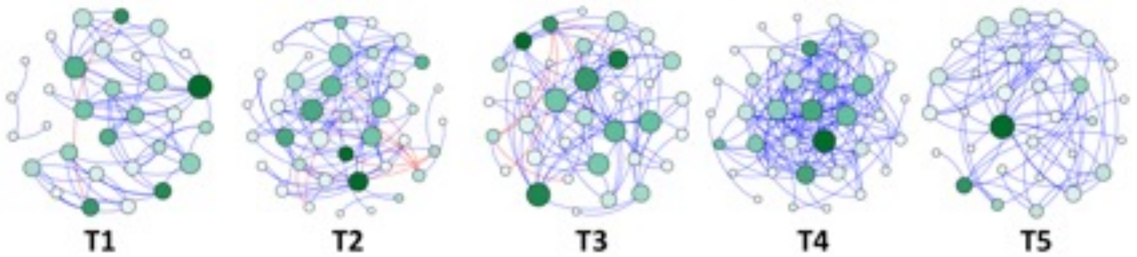

Supplementary Figure 4. Network co-occurrence analysis of archaeal community in soils treated with different rates of CTS. A connection stands for SparCC correlation with magnitude > 0.7 (positive correlation – blue edges) or < -0.7 (negative correlation – red edges) and statistically significant ($P < 0.01$). Each node represents taxa affiliated at OTU level based on 16S rRNA, and the size of a node is proportional to the number of connections (that is, degree). The color of the node is based on the values of betweeness centrality, where darker green indicates the nodes with highest values. Treatments: T1 = 0 ton ha^{-1} of CTS; T2 = 2.5; T3 = 5; T4 = 10; T5 = 20.
